# Supplementary material for: Carboxysome encapsulation of the CO2-fixing enzyme Rubisco in tobacco chloroplasts
Source: Nat Commun. 2018 Sep 3;9:3570. doi: 10.1038/s41467-018-06044-0 (PMC6120970; doi:10.1038/s41467-018-06044-0)
Supplement: Supplementary file 1 — Supplementary Information [file 41467_2018_6044_MOESM1_ESM.pdf]

Carboxysome encapsulation of the CO_2-fixing enzyme Rubisco in tobacco chloroplasts

Long *et al.*

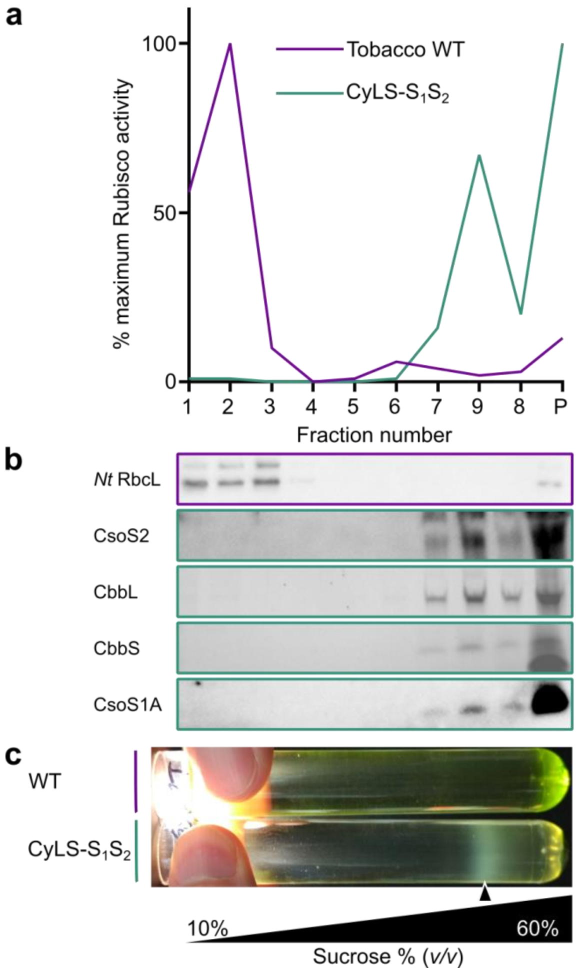

Supplementary Figure 1. Sucrose-gradient centrifugation of plant-derived carboxysomes.

Sucrose gradient purification of carboxysomes expressed in tobacco chloroplasts. Leaves of wild-type (WT) tobacco and CyLS-S_1S_2 plants were extracted as described in the Methods. A fraction representing insoluble material from both plants, containing carboxysome particles in the case of CyLS-S_1S_2, but only carry-over quantities of soluble Rubisco from wild-type, was applied to 10-60% (*w/v*) sucrose gradients and centrifuged (105,000 × g, 60 min). Fractions (1.5 mL) of each gradient were removed from the bottom of each tube, immediately above the pellet (P). The pellet was resuspended in residual buffer and collected separately. (**a**) Fixation of $^{14}CO_2$ by sucrose gradient fractions expressed as % maximum activity in each gradient. The magenta line indicates the profile of Rubisco activity for wild-type leaf extract and the cyan line indicates activity for CyLS-S_1S_2 extract. (**b**) Western blots for wild-type *Nicotiana tabacum* Rubisco LSU (*Nt* RbcL), *Cyanobium* CsoS2, and CsoS1A proteins, and Stain-free gel images indicating the location of *Cyanobium* CbbL and CbbS, present in each sucrose gradient fraction from (**a**). The border colour indicates the protein's association with the wild-type (magenta) or CyLS-S_1S_2 (cyan) gradient. Photograph of each gradient (**c**) with an arrowhead indicating the location of carboxysome particle band in the CyLS-S_1S_2 sample.

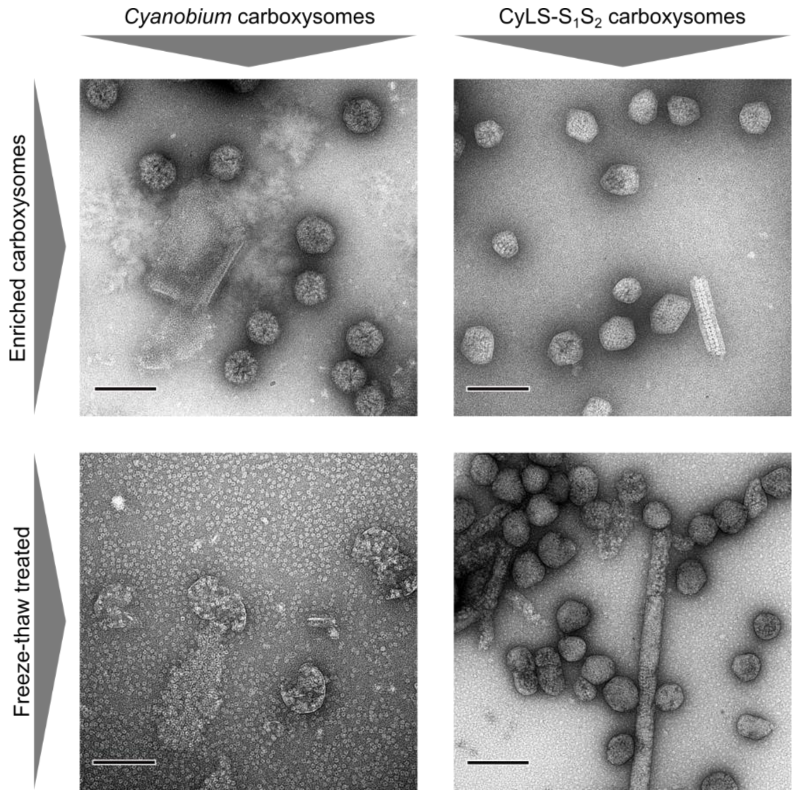

Supplementary Figure 2. Rubisco release from enriched carboxysomes.

Carboxysomes from both wild-type *Cyanobium* cells and CyLS-S_1S_2 plants were enriched to the step immediately prior to sucrose gradient purification according to the Methods. Note the relative purity of the CyLS-S_1S_2 particles. To release Rubisco, enriched fractions were pelleted by centrifugation (20,000 × g, 10 min) and pellets frozen at -20 °C for at least 30 min. Frozen pellets were rapidly resuspended in TEMB buffer to effect carboxysome breakage. Fractions prior to and after this treatment were imaged via transmission electron microscopy after negative staining. Note the freeze-thaw procedure was extremely successful for *Cyanobium* carboxysomes with free Rubisco and broken carboxysome shells clearly visible after treatment. CyLS-S_1S_2 carboxysomes, however, appeared resistant to breakage using this approach. The enriched carboxysomes from both sources are those used in Rubisco assays for intact carboxysomes. Freeze-thawed *Cyanobium* carboxysomes were used for assay of broken *Cyanobium* carboxysomes to evaluate the role of the shell in Rubisco catalysis. Scale bars are 200 nm.

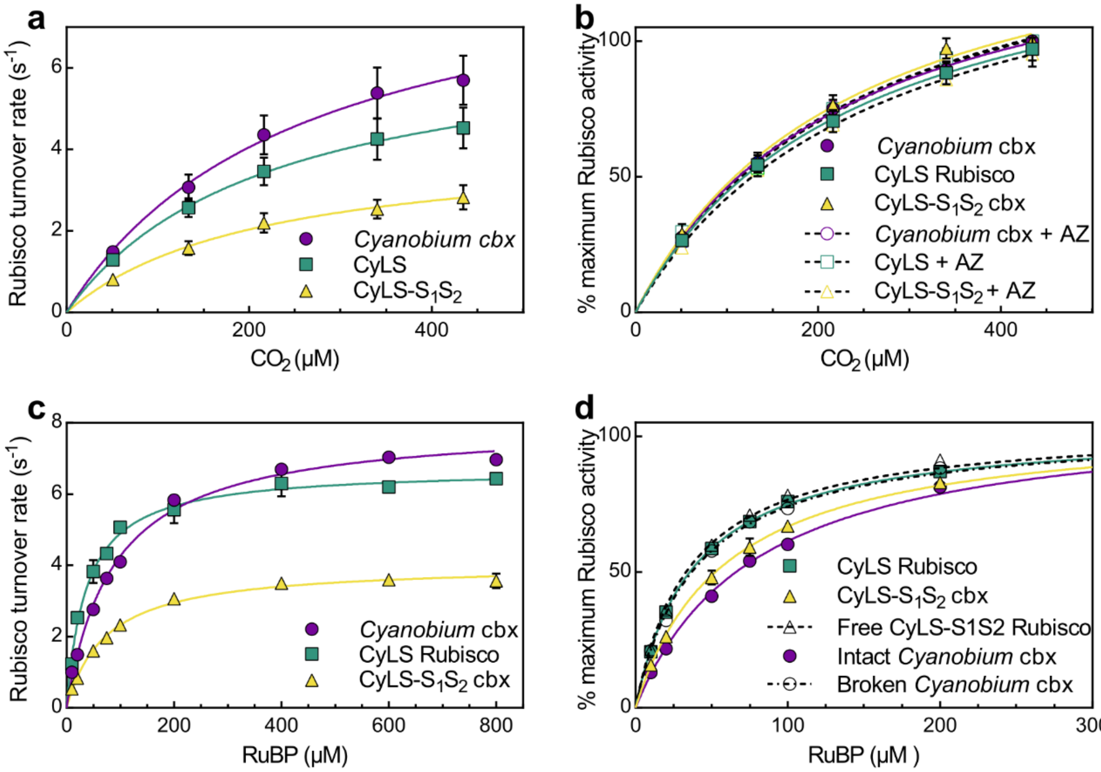

Supplementary Figure 3. CO_2 and RuBP response curves of carboxysomal and free Rubisco.

Typical CO_2 and RuBP response curves for Rubiscos analysed in this study. (**a**) CO_2 response of Rubisco from enriched fractions of wild-type *Cyanobium* carboxysomes, the free Rubisco from CyLS plants and that of isolated carboxysomes from CyLS-S_1S_2 plants. Data are plotted as turnover numbers (s^{-1}). Note that the CyLS-S_1S_2 carboxysome response has a significantly lower k_{cat}. (**b**) The same data as in (**a**) plotted on a percent of maximum rate basis, in addition to rates in the presence of the carbonic anhydrase inhibitor acetazolamide (AZ, 500 μM). Note here no change in CO_2 concentration at half maximum rates (K_C), regardless of the presence of a carboxysome shell or AZ. (**c**) RuBP response of Rubisco from enriched fractions of wild-type *Cyanobium* carboxysomes, the free Rubisco from CyLS plants and that of isolated carboxysomes from CyLS-S_1S_2 plants, plotted as turnover rates. Again, note the low maximum rate attained by CyLS-S_1S_2 carboxysomes. (**d**) The same data as in (**c**) plotted on a percent of maximum rate basis, in addition to rates obtained for free Rubisco from CyLS-S_1S_2 leaf homogenates after removal of insoluble carboxysome material by centrifugation, and rates for freeze-thaw broken *Cyanobium* carboxysomes. Note here that carboxysome-encapsulated Rubiscos (intact *Cyanobium* cbx and CyLS-S_1S_2 cbx) have higher $K_{M\mathrm{RuBP}}$ values than their naked counterparts, implying resistive influx of RuBP across intact carboxysome shells. In all cases error bars are ± s.e.m. (n = 3-6).

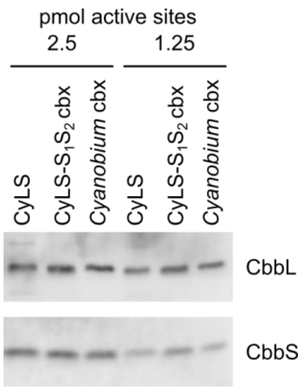

Rubisco source	Estimated stoichiometry	
	CbbS	CbbL
CyLS leaf extract	8.0	8.0
CyLS-S_1S_2 carboxysomes	8.9 ± 1.1	9.7 ± 1.8
Cyanobium carboxysomes	6.4 ± 0.7	8.6 ± 1.3

Supplementary Figure 4. Relative *Cyanobium* Rubisco subunit stoichiometry.

Rubisco active site concentrations in extracts of CyLS plants and isolated CyLS-S_1S_2 and *Cyanobium* carboxysomes were estimated using radiolabelled [^{14}C] carboxyarabanitol-P_2 as described in the Methods. Approximately equal quantities of Rubisco active sites from each preparation were then separated on SDS-PAGE gels and transferred to PVDF membranes prior to immuno-blotting for the large (CbbL) and small (CbbS) subunits of *Cyanobium* Rubisco. The analysis was carried out on a range of active site concentrations from approximately 1.25 – 10 pmol per lane (1.25 and 2.5 pmol are shown). Stoichiometries of each subunit were estimated for each quantity of active sites loaded onto the gel, relative to that found for CyLS Rubisco which was assumed to form L_8S_8 holoenzymes. Data are presented as the means of four active site concentrations ± s.d.

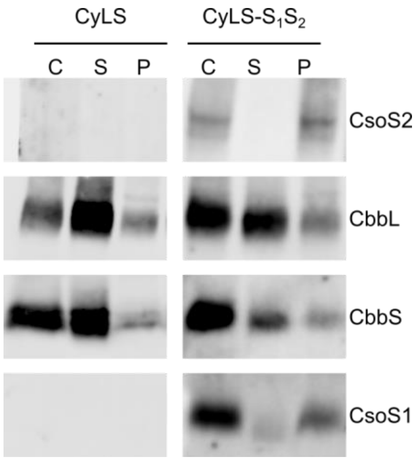

Supplementary Figure 5. Free and insoluble Rubisco in leaf homogenates.

Crude leaf homogenates (C) from both CyLS and CyLS-S_1S_2 plants were obtained by extracting leaf discs as described for leaf protein in the Methods. Extracts were centrifuged (20,000 × g, 20 min) and the supernatant fraction (S) removed to another tube. Pellet fractions (P) were resuspended to their original volume. Samples of each fraction were separated by SDS-PAGE and transferred to PVDF membranes prior to immuno-blot detection of CsoS2, CbbL, CbbS and CsoS1A. Note the extraction of significant quantities of CbbL and CbbS to the supernatant fraction in CyLS-S_1S_2 plants, indicating either a loss of Rubisco from carboxysomes during homogenization or a proportion of unpacked Rubisco exists in these plants. Comparable results were obtained with just a 1 min centrifugation step.

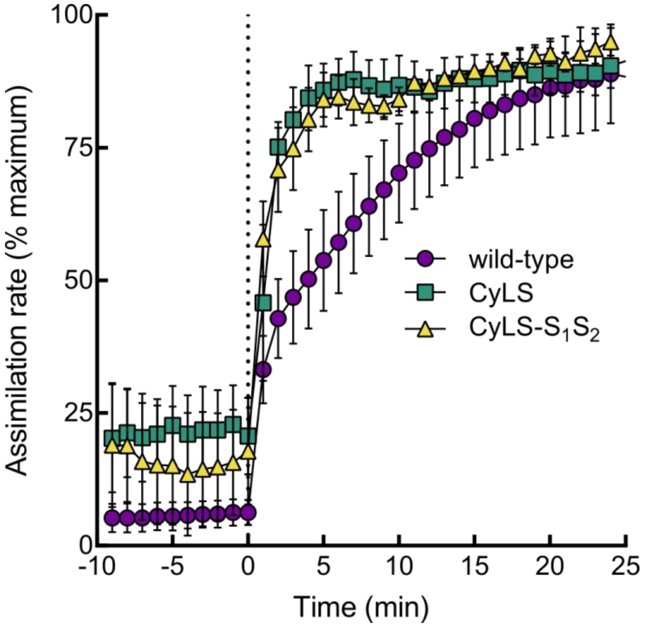

Supplementary Figure 6. Light activation of CO_2 assimilation in tobacco lines.

Assimilation rates from wild-type tobacco and transformant lines CyLS and CyLS-S_1S_2, normalized to maximum rates in each line. Leaves were dark acclimated for 10 minutes prior to illumination at 1500 μmol photons·m^{-2}·s^{-1}. CO_2 assimilation was monitored over the ensuing minutes via gas exchange under 400 μbar CO_2 at 25 °C. Note the relatively rapid attainment of maximum rates in both the CyLS and CyLS-S_1S_2 lines compared with wild-type tobacco. Rates are comparable to those at 400 μbar as presented in Fig. 5a,b. Data are presented as the means of measurements from three individual plants ± s.e.m.

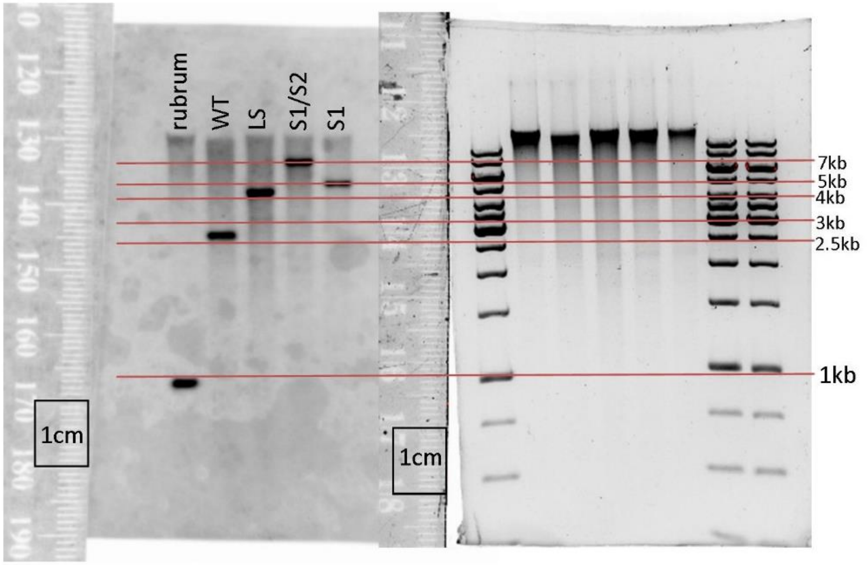

Supplementary Figure 7. Uncropped image of Southern blot relating to Figure 2.
Figure shows the Southern blot (left) and the original SYBRSafe-stained agarose gel (right).

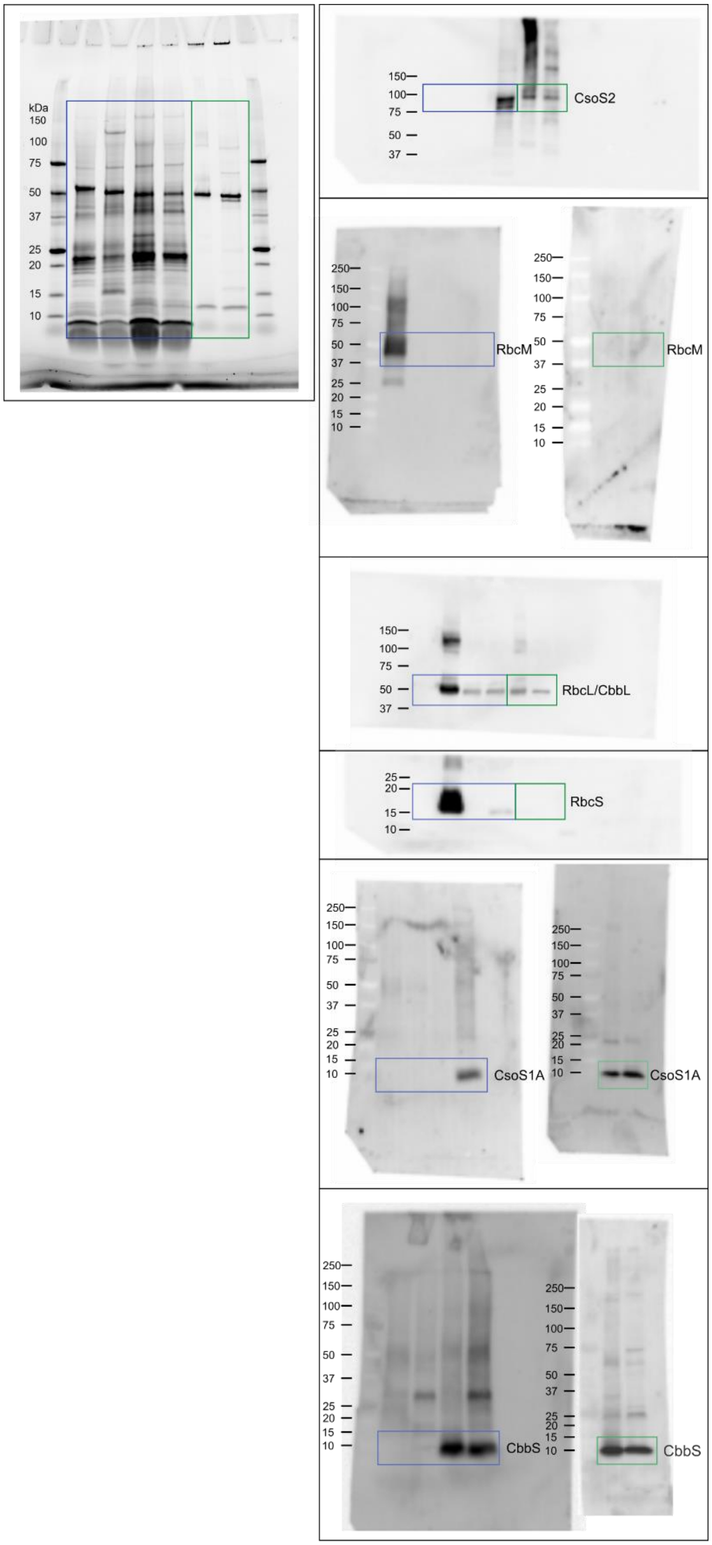

Supplementary Figure 8. Uncropped images of gel and western blots relating to Figure 3. Blue (Fig 3a) and green (Fig 3h) borders indicate cropping regions for use in figure construction.
